# Comparison of Fracture Resistance of 3D Printed and Milled Zirconia Veneers and Anterior Crowns

**DOI:** 10.1002/cre2.70440

**Published:** 2026-08-26

**Authors:** Les Kalman, Abdulaziz Alhothan, Mehrsima Ghavami‐Lahiji, Akimasa Tsujimoto

**Affiliations:** ^1^ Schulich School of Medicine & Dentistry Western University London Canada; ^2^ Department of Dental Health, College of Applied Medical Sciences King Saud University Riyadh Saudi Arabia; ^3^ Department of Restorative Dentistry, Dental Sciences Research Center, School of Dentistry Guilan University of Medical Sciences Rasht Iran; ^4^ Department of Operative Dentistry Aichi Gakuin University School of Dentistry Nagoya Japan; ^5^ Department of Operative Dentistry University of Iowa College of Dentistry Iowa City Iowa USA; ^6^ Department of General Dentistry Creighton University School of Dentistry Omaha Nebraska USA

**Keywords:** 3D‐printing, additive manufacturing, anterior crowns, CAD/CAM milling, fracture resistance, prosthodontics, restorative dentistry, zirconia

## Abstract

**Objectives:**

It has recently become possible to prepare monolithic zirconia restorations through 3D printing, in addition to the typical milling technique. In this study, the fracture resistance of anterior crowns and veneers prepared by both techniques was compared.

**Materials and Methods:**

Digital files were created for the crown and veneer, and restorations were created by milling (BruxZir Esthetic zirconia) and stereolithography 3D printing (LithaCon 3Y 210). The restorations were cemented to resin abutments and subjected to 50,000 thermal cycles before fracture resistance testing, measuring maximum load at fracture. Fracture resistance was evaluated using a universal testing machine. Data were statistically analyzed at a significance level of α = 0.05.

**Results:**

The milled crowns had values of 2318.37 ± 240.91 N, while the printed crowns showed 1988.15 ± 116.14 N (*p* = 0.009). The milled veneers had values of 1640.95 ± 173.19 N, and printed veneers of 1325.75 ± 130.71 N (*p* = 0.027).

**Conclusions:**

Clinically estimated occlusal forces in the anterior region are under 200 N, and so while printed restorations had significantly lower fracture resistance than milled ones, this was still far higher than the forces the materials are expected to encounter.

## 1 Introduction

Recently, the field of restorative dentistry has witnessed significant advancements in manufacturing techniques and materials. Zirconia has demonstrated a continued increase in popularity, especially as a material for restorative procedures due to its durability (Su et al. 2023), metal‐free composition (Refaie et al. 2023), physical properties (Lin et al. 2021), biocompatibility (Lin et al. 2021), and esthetics (Arslan and Deg˘irmenci 2024; Mazaheri et al. 2025).

Core substructures made of zirconia can be created and then covered with glass ceramics using layering or pressing. However, the moist oral environmental and masticatory forces may weaken the prosthesis and lead to cracking or chipping (Pieralli et al. 2018). To address this issue, monolithic zirconia dental restorations have been developed, featuring a full anatomic contour, eliminating the need for an extra veneer layer (Choi et al. 2017).

With ongoing technological advancements, the advent of CAD/CAM fabrication techniques and monolithic zirconia has expanded the applications for dental restorations that require minimal tooth preparation. These applications include partial coverage restorations, crowns, endocrowns, and bridges. Monolithic zirconia restorations are typically produced using a subtractive manufacturing process, which involves milling zirconia blocks or discs to fabricate the desired restoration. This method has become popular in dental practices due to its reliability and consistent outcomes (Refaie et al. 2023; Rues et al. 2023).

There are certain disadvantages to subtractive zirconia manufacturing, including the challenge of producing a restoration with intricate structures (i.e., deep grooves) and the difficulty of milling a thin margin without the risk of chipping (Rues et al. 2023; Abdulazeez and Majeed 2022). Furthermore, this process results in significant material waste (Alghauli et al. 2025), the need for frequent replacement of cutting tools, and high production costs (Alao et al. 2017).

Recently, 3D printing (3DP) or additive manufacturing (AM) has made significant advancements in the dental field, offering enhanced design flexibility and the potential for more cost‐effective production. 3DP constructs zirconia restorations layer by layer, enabling the creation of intricate geometries while minimizing material waste (Su et al. 2023; Branco et al. 2023).

3DP of zirconia presents a compelling alternative to subtractive manufacturing, particularly for anterior veneers and crowns, as it results in fewer marginal imperfections (Li et al. 2023). Furthermore, 3DP eliminates the need for drill compensation at sharp edges, a common limitation associated with traditional milling machines (Rues et al. 2023; Dahl et al. 2017).

Among the various zirconia 3DP techniques, stereolithography is utilized in many studies. Other techniques include binder jetting, material extrusion, direct energy deposition, and powder bed fusion (Galante et al. 2019). Stereolithography is particularly noteworthy for its ability to produce high‐quality surface finishes, making it ideal for aesthetic applications in dental restorations (Rues et al. 2023). It is currently the only available approach for fabricating fully anatomical 3DP zirconia crowns, offering exceptional precision and detail essential for achieving superior aesthetic results (Schweiger et al. 2021).

Vat polymerization techniques, such as stereolithography (SLA) and digital light processing (DLP), have shown strong potential for fabricating precise dental restorations. These methods produce zirconia “green bodies,” from which an acrylic binder is subsequently removed when they are sintered to achieve full density. While additively manufactured zirconia has demonstrated promising mechanical properties, the processing steps remain time‐consuming and may influence the final performance (Rues et al. 2023; Kalman and Tribst 2024; Revilla‐León et al. 2022).

The literature indicates that fracture is a primary cause of failure in dental ceramics under long‐term oral loading conditions. Previous studies have reported that 3D‐printed zirconia exhibits adequate fracture strength in biaxial testing of simplified disk specimens (Zenthöfer, Ilani et al. 2024; Rues et al. 2024). However, it remains unclear whether restorations with more complex geometries, such as veneers and crowns, demonstrate comparable fracture resistance to conventionally milled zirconia.

Fracture resistance is a critical factor for the durability and clinical success of anterior restorations, which are subjected to both functional and esthetic demands. This property is influenced by multiple factors, including surface characteristics, material properties, and fabrication techniques.

To date, research on the fracture resistance of 3DP zirconia restorations has primarily focused on posterior full crowns and occlusal veneers (Refaie et al. 2023; Zenthöfer, Fien et al. 2024). However, 3DP zirconia is increasingly being utilized in anterior restorations. In this study, we explored the fracture resistance of both milled and 3DP anterior crowns and veneers following thermal cycling, providing valuable insights into their performance in these aesthetically and functionally demanding applications.

The null hypothesis was that there would be no significant difference in fracture resistance between anterior crowns and veneers produced using milling techniques and those created through 3DP.

## 2 Materials and Methods

### 2.1 Prostheses Fabrication

**Figure 1:**
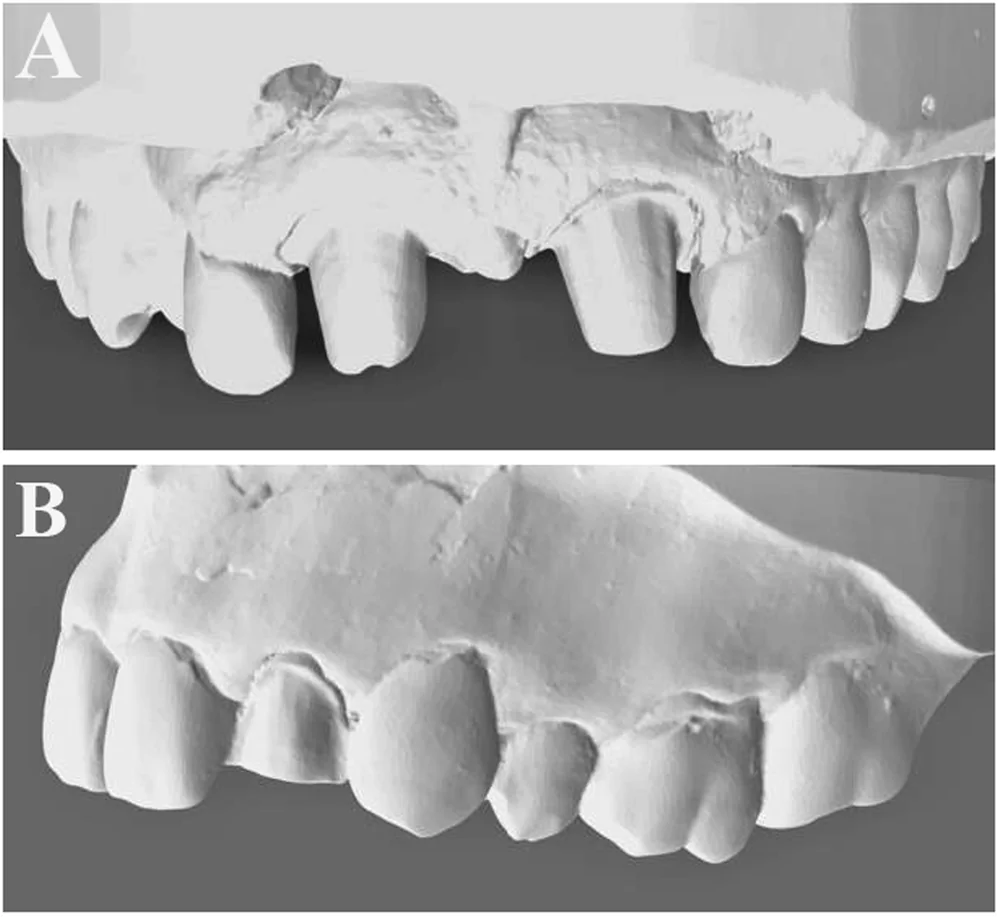
Virtual model of central incisors prepared for full ceramic crown coverage (A). Virtual model of a lateral incisor prepared for a ceramic veneer (B).

Two demonstration casts were used to fabricate specimens. Cast 1 included both maxillary central incisors prepared for full‐coverage ceramic crowns, and Cast 2 included a maxillary lateral incisor (Tooth 22) prepared for a ceramic veneer (Figure 1). Digital impressions of both casts were obtained using a laboratory scanner (Straumann CARES 3, Straumann, Basel, Switzerland), and the corresponding STL files were generated for subsequent CAD/CAM and 3DP workflows.

**Figure 2:**
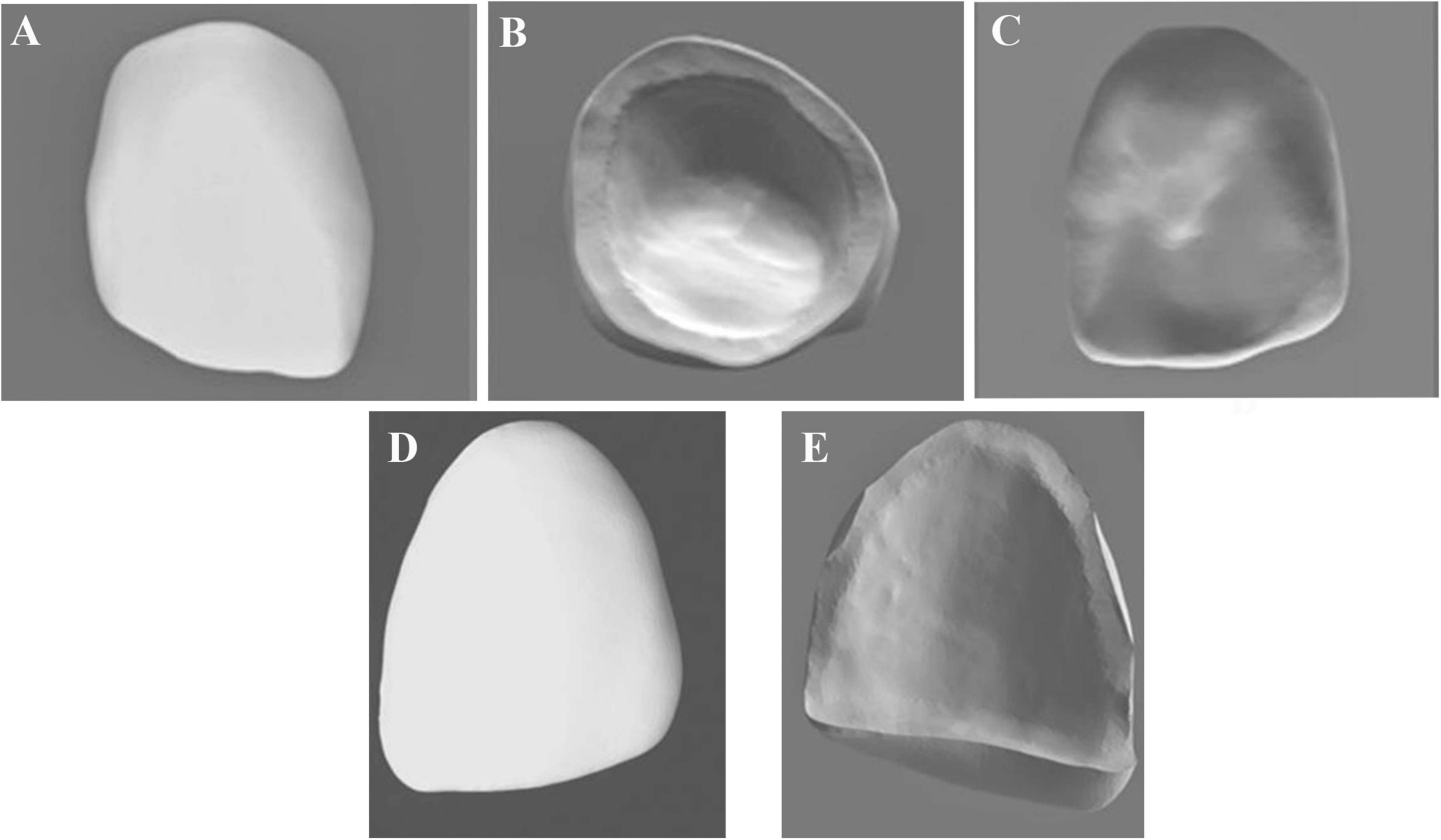
Digital design of anterior crown (#11), facial (A), internal (B), and lingual (C) view. Digital design of anterior veneer (#22), facial (D), and internal (E) view.

For Cast 1, full‐contour zirconia crowns were virtually designed for each central incisor, and for Cast 2, a zirconia veneer was designed for the prepared lateral incisor (Figure 2). All digital designs were performed using dental CAD software (Exocad GmbH, Darmstadt, Germany) by a certified dental technician at Schulich Dentistry to standardize morphology and contour across all specimens.

**Figure 3:**
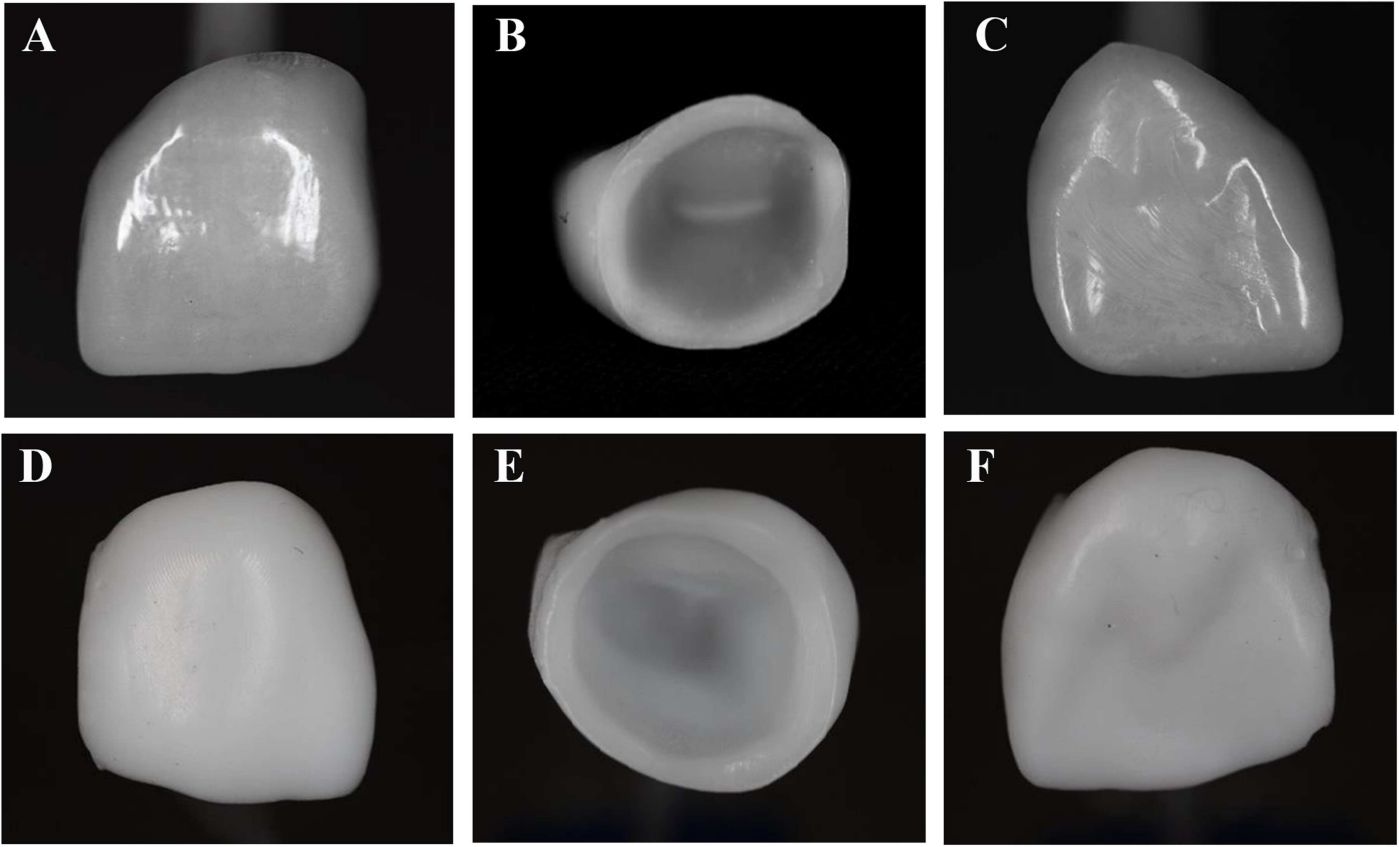
Milled anterior zirconia crown (#21): Facial, intaglio, and palatal views, respectively (A–C). 3DP anterior zirconia crown (#11): Facial, intaglio, and palatal views, respectively (D–F).

The STL file for Cast 1 was transmitted electronically to a commercial dental laboratory (Glidewell, Newport Beach, USA) for fabrication of six milled monolithic zirconia crowns corresponding to the prepared left central incisor (Tooth #21). The same STL file was also sent to a ceramic 3DP manufacturer (Lithoz, Vienna, Austria) for the fabrication of six zirconia crowns for the contralateral central incisor (Tooth #11) using a lithography‐based ceramic manufacturing process (Figure 3). Zirconia crowns in the 3D‐printed group were fabricated using a stereolithography‐based 3D printing system (Cerafab, Lithoz, Austria) and a commercially available zirconia slurry (LithaCon 3Y 210, Lithoz, Austria). After printing, the green bodies had the binder removed and were sintered according to the manufacturer's instructions to achieve full density.

**Figure 4:**
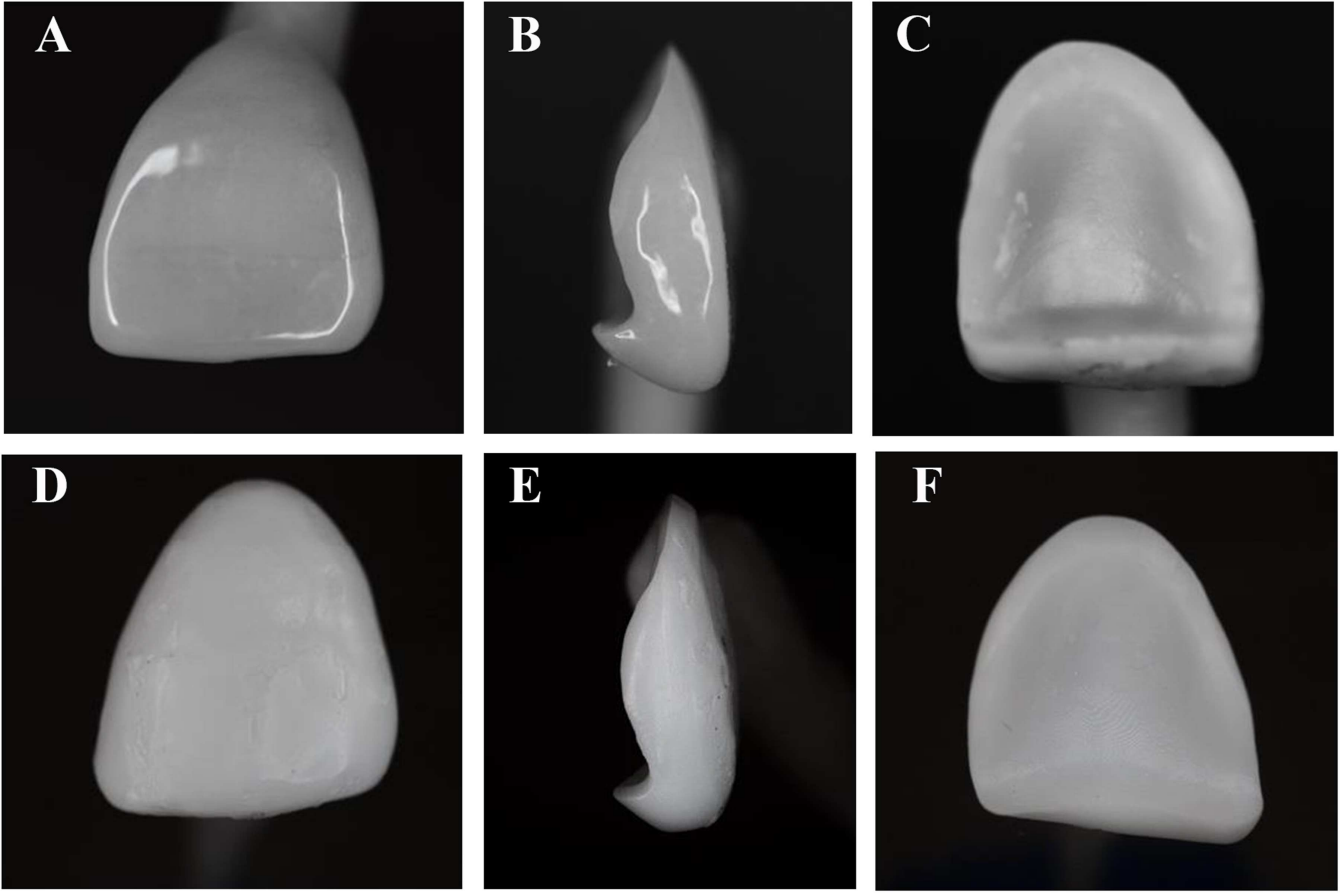
Milled anterior zirconia veneer (#22): Facial, proximal, and intaglio views, respectively (A–C). 3DP anterior zirconia veneer (#22): Facial, proximal, and intaglio views, respectively (D–F).

Similarly, the veneer design for Cast 2 was exported as an STL file and submitted to Glidewell for fabrication of six milled monolithic zirconia veneers for Tooth #22. For comparison, the same file was sent to Lithoz to produce six 3D‐printed zirconia veneers (Figure 4).

The STL files that were electronically transmitted to a commercial dental laboratory contained no instructions to modify the original digital design. However, upon delivery, it was evident that minor design edits had been implemented. The crowns and veneers were fabricated by milling BruxZir Esthetic zirconia, for which the manufacturer reports an average flexural strength of approximately 870 MPa (Glidewell 2025). In addition, the specimens were stained and glazed by the laboratory as part of their routine workflow before being returned for in vitro evaluation.

In contrast, the STL files provided to the ceramic printing company (Lithoz, Austria) were printed without digital adjustment. The 3DP crowns and veneers were produced using a lithography‐based ceramic manufacturing technology (CeraFab 7500, Lithoz) and a 3 mol% yttria‐stabilized zirconia slurry (LithaCon 3Y 210; Lithoz, Vienna, Austria), with a reported three‐point bending strength of 770 MPa, and four‐point bending strength of approximately 1000 MPa and a surface roughness (Ra) below 1.0 μm (Lithoz 2025, 2026).

Printing was performed with an in‐plane pixel size of 40 μm and a layer thickness of 25 μm, followed by debinding and sintering. Linear sintering shrinkage of approximately 20% was compensated for by the manufacturer's preprocessing software, which automatically scaled the STL designs prior to printing to achieve the intended final dimensions.

### 2.2 Fracture Resistance Testing

The virtual models were used for abutment tooth fabrication, for both veneers and crowns, using resin‐based 3D printing (Model Resin, FormLabs, Somerville, MA, USA), with an in‐lab 3D printer (FormLabs 3, FormLabs). The abutment surfaces and the intaglio surfaces of the veneers and crowns were air‐abraded with aluminum oxide particles, followed by ultrasonic cleaning and air‐drying. Air abrasion was performed using 50 μm Al_2_O_3_ particles at a pressure of 2.5 bar for 10 s at a distance of 10 mm. After air abrasion, specimens were ultrasonically cleaned in distilled water for 5 min and air‐dried.

The intaglio surfaces were treated with a ceramic primer (Ceramic Primer Plus, Kuraray Noritake Dental, Tokyo, Japan) and cemented using a resin luting cement according to the manufacturer's instructions (Panavia V5, Kuraray Noritake Dental). Light curing was performed using an LED curing unit (Valo, Ultradent Products, South Jordan, UT, USA) from several different angles for 20 s per surface. During cementation, a standardized load of 10 N was applied for 5 min to ensure uniform seating of all restorations. All the bonded specimens were then immersed in distilled water at 37°C for 24 h.

**Figure 5:**
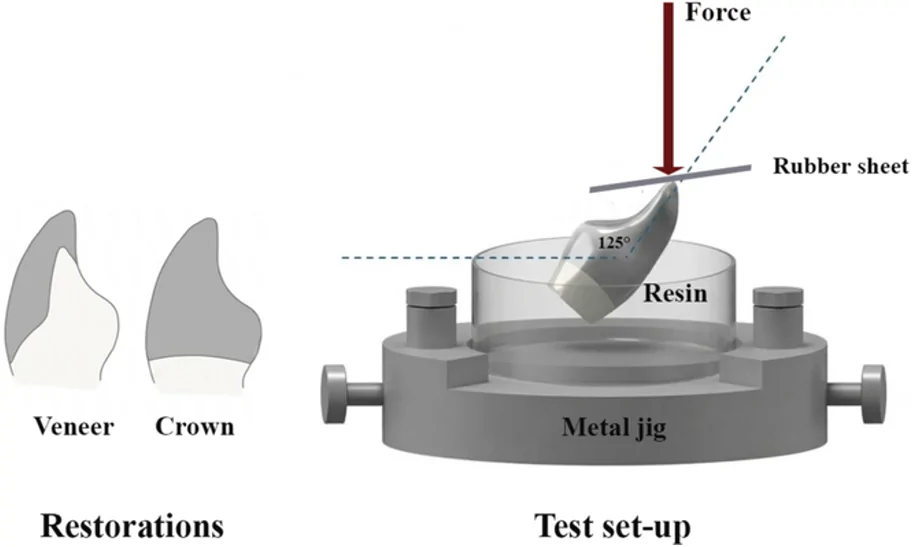
Representative designs of the anterior ceramic restorations evaluated in this study: Ceramic veneer and full‐coverage ceramic crown (left). Schematic illustration of the experimental set‐up used for fracture resistance testing (right).

Both the milled and 3D‐printed prostheses were subjected to thermocycling for 50,000 cycles to simulate approximately 5 years of clinical service (Gale and Darvell 1999). Thermal cycling was performed between 5°C and 55°C, with a dwell time of 30 s in each bath and a transfer time of 10 s, following the aging relationship proposed by Gale and Darvell (Gale and Darvell 1999). Following thermocycling, cemented specimens were embedded in self‐curing acrylic resin up to 2 mm below the cementoenamel junction (CEJ) (Lin et al. 2012; Jurado et al. 2025), with the long axis of each specimen oriented at 125° relative to the base of a prefabricated mold (Lin et al. 2012) (Figure 5).

Each specimen was placed on a jig and subjected to compressive loading until fracture using a universal testing machine (Instron 5965, Norwood, MA, USA) equipped with Bluehill software (Version 3.22.1373) and a 5 kN load cell. The loading apparatus consisted of a stainless‐steel plunger with a 5‐mm‐diameter spherical tip. During testing, a 1.5 mm rubber sheet was interposed between the indenter and the incisal edge to simulate a food bolus, distribute the applied load, and reduce stress concentration at the loading contact (Jurado et al. 2025). Load was applied at a crosshead displacement rate of 1 mm/min, and the maximum force (N) at the point of failure was recorded for each specimen.

All statistical analyses were performed using SPSS Version 28 (IBM, New York, USA) at a significance level of *α* = 0.05. Normality of data distribution was assessed using the Shapiro–Wilk test, and homogeneity of variances was evaluated using Levene's test. An independent‐samples *t*‐test was used to compare maximum fracture load values between the milled and 3DP groups.

## 3 Results

Table 1 presents overall descriptive statistics for the milled and 3DP crown groups, including mean ± standard deviation, and minimum and maximum values for each group. The mean maximum load of the 3DP crowns (1988.16 ± 116.14 N) was significantly lower than that of the milled crowns (2318.37 ± 240.90 N) (*p* = 0.009).

**Table 1:** Maximum load at fracture resistance test (*N*) for milled and 3DP crowns.

Crowns						
	*N*	Mean	SD	Min	Max	*p* value
Milled crowns	6	2318.37	240.91	1864.40	2550.20	*p* = 0.009
3DP crowns	6	1988.15	116.14	1850.42	2157.65	

Table 2 presents overall descriptive statistics for the milled and 3DP veneer groups, including mean ± standard deviation and minimum and maximum values for each group. The mean maximum load of 3DP veneers (1325.75 ± 130.71 N) was significantly lower than that of the milled veneers (1640.95 ± 173.19 N) (*p* = 0.027).

**Table 2:** Maximum load at fracture resistance test (*N*) for milled and 3DP veneers.

Veneers						
	*N*	Mean	SD	Min	Max	*p* value
Milled veneers	6	1640.95	173.19	1450.81	1935.71	*p* = 0.027
3DP veneers	6	1325.75	130.71	1112.21	1482.19	

Overall, the milled zirconia crowns and veneers showed a significantly higher fracture resistance compared with their 3DP counterparts.

## 4 Discussion

The null hypothesis, that there would be no significant difference in fracture resistance between 3DP and milled zirconia anterior crowns and veneers, was rejected.

In this study, 3DP standard resin was utilized to replicate abutment teeth for evaluating and comparing the fracture strength of ceramic restorations fabricated by two approaches (Refaie et al. 2023). This approach is informed by previous research, which has assessed the fracture strength of full ceramic restorations using various materials, including cobalt–chromium (CoCr), fiber‐reinforced composite, stainless steel, acrylic resin, epoxy resin, and dentin (Zenthöfer, Fien et al. 2024; Ioannidis et al. 2020; Åkesson et al. 2009). It has been reported that the stress distributions in dentin and epoxy resin more accurately simulate clinical conditions, providing realistic fracture strength values compared to stainless steel and copper, closely mimic the mechanical properties and stress responses of natural dentin (Yucel et al. 2012).

In addition to the mechanical properties of the abutment substrate, the bonding interface may also influence the fracture behavior of adhesively luted ceramic restorations. In the present study, zirconia restorations were air‐abraded and treated with an MDP‐containing ceramic primer prior to cementation with Panavia V5 resin cement. The 10‐MDP in the primer is a functional phosphate monomer that has been shown to establish durable chemical bonding with zirconia, while airborne‐particle abrasion enhances micromechanical retention (Koko et al. 2022). The 3D‐printed Model Resin abutments are compatible with methacrylate‐based resin cement systems, providing a standardized and reproducible adhesive interface. Although the bonding characteristics of resin‐based abutments differ from those of natural dentin, the same substrate, surface treatment, and cementation protocol were applied to all specimens, thereby minimizing variability related to the adhesive interface and allowing comparison of the manufacturing methods.

Accelerated aging tests offer crucial insights into ceramic dental restorations’ long‐term performance and lifetime predictions. In the present study, an aging protocol was conducted to mimic the fluctuating temperature conditions encountered in the oral environment. Based on the relationship proposed by previous studies, in which 10,000 thermocycles were suggested to approximate 1 year of clinical service, the 50,000‐cycle protocol used in this study may be considered representative of approximately 5 years of hydrothermal aging in the oral environment (Gale and Darvell 1999; Amir Rad et al. 2015). This information is essential for understanding the long‐term clinical performance and expected lifespan of these ceramic dental prosthetics, providing valuable guidance for clinicians and patients. However, it should be noted that thermocycling alone does not fully reproduce the complex oral environment, which also involves cyclic masticatory loading as well as chemical challenges such as pH fluctuations, moisture exposure, and hydrolytic degradation.

It has been reported that the maximum biting force in the anterior region is significantly lower than in the posterior region, with males exerting approximately 176 N and females about 108 N. These values are much lower than the forces in the posterior teeth, which range from 450 to 520 N and can increase to 790 N in cases of bruxism (Nishigawa et al. 2001). While bruxism can elevate forces on anterior teeth, they remain generally lower than those in the posterior region. Our findings showed that the fracture resistance of crowns after hydrothermal cycling was 2318.37 ± 240.91 N for milled crowns and 1988.156 ± 116.14 N for 3DP crowns (Table 1). These values far exceed the maximum forces typically encountered in bruxism. Similarly, the fracture resistance of veneers was 1640.95 ± 173.19 N for milled veneers and 1325.75 ± 130.71 N for 3DP veneers (Table 2), again surpassing the maximum biting forces in the anterior region. Consequently, crowns and veneers fabricated using both milling and 3DP techniques demonstrate enough fracture resistance to support even the increased forces associated with bruxism, and even after simulated aging.

In the current study, milled zirconia crowns demonstrated significantly higher fracture resistance compared to their 3DP counterparts. These findings align with the results reported by Refaie et al. (2023) and Zhai et al. (2025). However, they contrast with the observations made by Ioannidis et al. (2020). The same tendency was seen in the veneers. The differences in thickness and the functional demands placed on occlusal veneers versus anterior tooth veneers may have contributed to the contrasting findings between our study and those of earlier studies (Cherukara et al. 2002; Sirous et al. 2022). The difference can be explained by the fact that the thickness is often more consistent across the entire occlusal veneer surface, and this uniform thickness distribution helps distribute occlusal stresses evenly and prevent localized areas of weakness or vulnerability. It is important to note that those previous studies primarily focused on posterior crowns or occlusal veneers, whereas our research specifically examined anterior crowns and veneers. Similarly, Sasany et al. (2025) reported comparable trueness and retention between additively and subtractively manufactured zirconia laminate veneers, with no significant difference in debonding force. Their findings further support the clinical potential of additively manufactured zirconia restorations.

The lower fracture resistance observed for the 3D‐printed zirconia restorations may be related to differences in the manufacturing process; however, the present study was not designed to identify the underlying mechanisms. Previous studies have suggested that additive manufacturing may result in greater residual porosity than conventional milling, which could influence the mechanical performance of zirconia restorations (Su et al. 2023; Refaie et al. 2023). Likewise, the highly viscous zirconia slurry used during the printing process has been reported to increase the likelihood of air entrapment, potentially creating defects that may act as stress concentrators under loading (Harrer et al. 2017). In addition, differences in the manufacturing process may produce distinct microstructures. For example, Yoo et al. (2023) reported a more heterogeneous grain structure and increased porosity in printed zirconia compared with milled zirconia, which was associated with differences in hardness and flexural properties. Furthermore, the layer‐by‐layer fabrication process has been proposed to introduce interlayer defects that could influence crack initiation and propagation (Zheng et al. 2020). However, because no fractographic, dimensional, or microstructural analyses were performed in the present study, these proposed mechanisms are speculative. They cannot be confirmed and should be regarded as possible explanations rather than definitive causes of the observed differences in fracture resistance.

One point to consider is that the fracture resistance of anatomically shaped, cemented restorations is influenced not only by the intrinsic flexural strength of the material but also by factors such as restoration geometry, manufacturing‐related defects, internal fit, cementation, and stress distribution during loading.

To date, anterior crowns and veneers fabricated from 3DP zirconia have shown promising outcomes in restoration design and in achieving precise marginal and internal fit (Rues et al. 2023; Kalman and Tribst 2024; Jahangiri et al. 2024). In this study, the fracture resistance of the printed crowns and veneers, while lower than that of their milled counterparts, still exceeded the forces expected in clinical use. However, the partial coverage of the abutment and the thinner wall thickness of veneers, compared to crowns, resulted in reduced fracture resistance, although they still comfortably exceeded the clinically estimated biting forces.

It is worth noting that the comparison between milled and 3D‐printed zirconia in the present study was conducted using commercially available zirconia materials, each specifically developed for its respective fabrication technique. As no single zirconia material is currently available that can be used for both milling and 3D printing, the influence of the manufacturing method cannot be completely separated from that of material composition. Therefore, the findings should be interpreted as reflecting the overall performance of the currently available additive and subtractive manufacturing workflows rather than the manufacturing process alone. As this study only investigated two materials, it is not clear how broadly the results generalize to milled and printed zirconia. In addition, fracture resistance was measured using a single‐load‐to‐fracture test after thermocycling, without incorporating cyclic preloading, which more closely replicates clinical masticatory fatigue. Further work with a wider range of materials, larger sample sizes, and combined thermo‐mechanical aging protocols would provide a more comprehensive assessment of the long‐term performance of these restorations.

An additional limitation is that the milled zirconia restorations were stained and glazed by the commercial laboratory, despite our request for uncharacterized specimens, whereas the 3D‐printed group did not undergo the same surface characterization. This uncontrolled modification may have introduced a potential confounding factor in the comparison. However, a recent in vitro study by Fiorin et al. (2025) on monolithic zirconia crowns showed that staining and glazing do not significantly affect survival probability or fracture resistance under fatigue loading compared with untreated controls. Therefore, although the effect of this surface treatment on fracture behavior is likely limited, it should still be considered when interpreting the present findings. Another limitation is that the milled restorations were returned with minor design modifications to their external morphology introduced by the laboratory, despite our request to preserve the original STL design. Because these alterations were beyond our control, they may have introduced additional variability in the comparison between groups. Furthermore, the use of contralateral maxillary central incisors from the same virtual model minimized anatomical variability between groups; however, minor anatomical differences cannot be completely excluded.

## 5 Conclusion

This study shows that the fracture resistance of monolithic milled zirconia crowns and veneers is significantly higher than that of their 3D‐printed counterparts. However, the fracture resistance of printed restorations still far exceeds the expected occlusal forces, even in cases of parafunction. The fracture resistance was measured after simulated aging, suggesting that the materials would be durable under clinical conditions. These results suggest that 3D‐printed zirconia has potential as a material for dental restorations.

## Author Contributions

**Les Kalman:** conceptualization, project administration, supervision, review and editing. **Abdulaziz Alhothan:** methodology, investigation, interpretation of data, writing – original draft, visualization. **Mehrsima Ghavami‐Lahiji:** methodology, investigation, interpretation of data, writing – original draft, review and editing. **Akimasa Tsujimoto:** conceptualization, methodology, investigation, interpretation of data. All authors read and approved the final manuscript.

## Conflicts of Interest

The authors declare no conflicts of interest.

## Data Availability

The data that support the findings of this study are available on request from the corresponding author. The data are not publicly available due to privacy or ethical restrictions.
